# Note on some antlions from Mozambique (Neuroptera: Myrmeleontidae)

**DOI:** 10.3897/BDJ.2.e1050

**Published:** 2014-02-03

**Authors:** Agostino Letardi

**Affiliations:** † Technical Unit for Sustainable Development and Innovation of Agro-Industrial System, ENEA, Rome, Italy

**Keywords:** Southeastern Africa, Myrmeleontidae, *
Jaya
*, *
Myrmeleon
*, *
Cueta
*, *
Macronemurus
*

## Abstract

Faunal data concerning 4 poorly known species from Southern Mozambique are reported. *Myrmeleon lanceolatus* Rambur, 1842 is reported for the first time from Mozambique.

## Introduction

Myrmeleontidae (antlions) is one of the largest families of Neuropterida, with more than 1500 species currently recognised by Stange (2004). This family includes some of the largest lacewings, with wingspan wingspans up to 16–17 cm (Mansell 1999). The richest species diversity of Myrmeleontidae tends is present in the semi-arid world areas and the southern part of Africa harbours a very high diverse fauna of antlions; in this region antlions represent an important element of the local insect fauna, with several endemic taxa (Mansell 1996). Despite this, faunal studies of African antlions are scarce and limited to specific areas (southern Africa, western Africa, Tunisia) and specific groups of antlions (mainly on the visually striking subfamily Palparinae): the latest general overview of African antlions goes back to Banks (1911). A more detailed review of African faunal studies for antlions is in Stange (2004). Recently, long term revisions of the fauna of West and South Africa are in progress (Mansell, Michel, pers. comm.) and that several genera or tribe not belonging to Palparinae were already reviewed (Mansell 1985, Mansell 1987, Michel and Akoudjin 2011, Michel and Akoudjin 2012, Prost 1996).

This scarce attention to antlions of African fauna is certainly due to the difficult to identify many of the genera and species because of their unresolved systematics. This situation can be attributed mainly to the numerous and intricate publications of the Catalan Jesuit priest L. Navás, containing a variety of scattered records and cryptic new descriptions (Monserrat 1986).

The aim of this short note is to contribute to the faunal the knowledge concerning African antlions, regarding very poorly known species.

## Materials and methods

All specimens have been collected at light trap in the same locality: only very few antlions have been preserved, because they have been not the target of the study. The dried pinned specimens are preserved in the CNBFVR (Centro Nazionale per lo Studio e la Conservazione della Biodiversità Forestale “Bosco Fontana” di Verona, Sede di Bosco Fontana. Marmirolo (Mantua), Italy) collection.

## Taxon treatments

### Jaya dasymalla

(Gerstaecker, 1863)

#### Materials

- **Type status:** Other material. **Occurrence:** recordedBy: P. Cerretti, D. Birtele, A. Campanaro; individualCount: 1; sex: female; **Location:** country: Mozambique; verbatimLocality: Matutuine, distr. Tinti Gala Lodge; verbatimLatitude: 26°38'44.3"S; verbatimLongitude: 32°50'25.1"E; **Event:** samplingProtocol: light trap; eventDate: 31.I.2008; **Record Level:** institutionCode: CNBFVR

#### Distribution

An antlion is widespread in Africa (Mansell, pers. com; Oswald 2013), particularly in south-eastern part of Africa, from Tanzania to South Africa (Mozambique included) according to Prost (1996); in Stange (2004), this taxa was reported in Kenya, Tanzania and Namibia; recently this species has been reported also in a generical forestry coastal area of North-eastern Mozambique (Pascal 2011). Nevertheless I was able to find in literature only some specimens cited for a precise locality (in the Illustrated database of African Neuroptera (http://www.africamuseum.be/collections/browsecollections/naturalsciences/biology/neuroptera/collection), 37 localities are reported, mostly from South Africa and a very few from Somalia): the present female specimen is the first cited for a precise locality in Mozambique.

### Myrmeleon lanceolatus

Rambur, 1842

#### Materials

- **Type status:** Other material. **Occurrence:** recordedBy: P. Cerretti, D. Birtele, A. Campanaro; individualCount: 1; sex: male; **Location:** country: Mozambique; verbatimLocality: Matutuine, distr. Tinti Gala Lodge; verbatimLatitude: 26°38'44.3"S; verbatimLongitude: 32°50'25.1"E; **Event:** samplingProtocol: light trap; eventDate: 31.I.2008; **Record Level:** institutionCode: CNBFVR

#### Distribution

According to Stange (2004), this species is distributed in Namibia and South Africa. There are several localities reported in online catalogues (in the Illustrated database of African Neuroptera, 16 localities are reported, mostly from South Africa and a very few from Namibia and Lesotho; four localities from western part of South Africa are reported in GBIF (2013); generically present also in Niger and Sierra Leone according to Oswald (2013); but the present male specimen is the first cited for Mozambique.

### Cueta mysteriosa

(Gerstaecker, 1893)

#### Materials

- **Type status:** Other material. **Occurrence:** recordedBy: P. Cerretti, D. Birtele, A. Campanaro; individualCount: 1; sex: female; **Location:** country: Mozambique; verbatimLocality: Matutuine, distr. Tinti Gala Lodge; verbatimLatitude: 26°38'44.3"S; verbatimLongitude: 32°50'25.1"E; **Event:** samplingProtocol: light trap; eventDate: 31.I.2008; **Record Level:** institutionCode: CNBFVR

#### Distribution

An antlion is very widespread in sub-Saharan Africa (Stange 2004, Oswald 2013), from Ivory Coast and Somalia to South Africa (in the Illustrated database of African Neuroptera, 37 localities are reported, mostly from eastern area of South Africa, some from Kenya; in GBIF (2013) is reported a locality from Tanzania; other 6 localities from Somalia, Kenya and Tanzania are reported in the database of Information System ZInsecta (http://www.zin.ru/projects/ZInsecta/eng/ZInsecta.asp). Two species of the same genus (*Cueta mosambica* and *Cueta heynei*), likely synonyms of *Cueta mysteriosa* (Mansell, per. comm.), have been cited in Mozambique (Navás 1914, Navás 1915), and recently (as Cueta cf. mysteriosa) the taxon has been reported for a generical forestry coastal area of North-eastern Mozambique (Pascal 2011), so the present report is the first for a precise locality of Mozambique after more than a century.

### Macronemurus tinctus

Kolbe, 1897

#### Materials

- **Type status:** Other material. **Occurrence:** recordedBy: P. Cerretti, D. Birtele, A. Campanaro; sex: 1 male, 1 female; **Location:** country: Mozambique; verbatimLocality: Matutuine, distr. Tinti Gala Lodge; verbatimLatitude: 26°38'44.3"S; verbatimLongitude: 32°50'25.1"E; **Event:** samplingProtocol: light trap; eventDate: 31.I.2008; **Record Level:** institutionCode: CNBFVR

#### Distribution

According to Oswald (2013) this species is distributed in Kenya, Angola, Mozambique, Tanzania, and Uganda. There are several localities reported in online catalogues (in the Illustrated database of African Neuroptera, 111 localities are reported, mostly from South Africa and Namibia, some from Zimbabwe, a couple from central area of Mozambique, a couple from Malawi, another from Zambia; two localities from South Africa are reported in GBIF (2013); another locality from Ethiopia is reported in the database of Information System ZInsecta). The present report is the third locality for Mozambique, the first in the southern part of this country.

## Supplementary Material

XML Treatment for Jaya dasymalla

XML Treatment for Myrmeleon lanceolatus

XML Treatment for Cueta mysteriosa

XML Treatment for Macronemurus tinctus
